# Assay of ethanol and congener alcohols in serum and beverages by headspace gas chromatography/mass spectrometry

**DOI:** 10.1016/j.mex.2021.101563

**Published:** 2021-10-29

**Authors:** Cora Wunder, Werner Pogoda, Alexander Paulke, Stefan W. Toennes

**Affiliations:** Department of Forensic Toxicology, University Hospital, Goethe University, Institute of Legal Medicine, Frankfurt/Main, Germany

**Keywords:** Post-offence drinking, Alcohol, Methanol, 1-propanol, Method validation, HS, headspace, GC, gas chromatography, MS, mass spectrometry, FID, flame ionization detection, ISTD, internal standard, Cal, calibrator, QC, quality control, GTFCh, Gesellschaft für Toxikologische und Forensische Chemie, EI, electron impact ionization, LOD, limit of detection, LLOQ, lower limit of quantitation, CV, coefficients of variation

## Abstract

The analysis of ethanol and of its congeners in blood plays an important role in forensic cases, especially when allegations are made that alcohol has been consumed after an accident. In alcoholic beverages, congener alcohols are by-products and are generated during fermentation. The assay of these compounds in serum samples and beverages has been previously performed using headspace-gas chromatography-flame ionization detection methods (HS-GC-FID). As an alternative, a robust headspace-gas chromatography-mass spectrometry (HS-GC-MS) procedure was developed and validated, which has the following advantages:

• Simultaneous determination of ethanol, congener alcohols and other endogenous substances.
• Reduction of matrix interference by increasing selectivity and specificity.
• Clear separation of the positional isomers 3-methyl-1-butanol and 2-methyl-1-butanol.

## Graphical abstract

**Figure:**
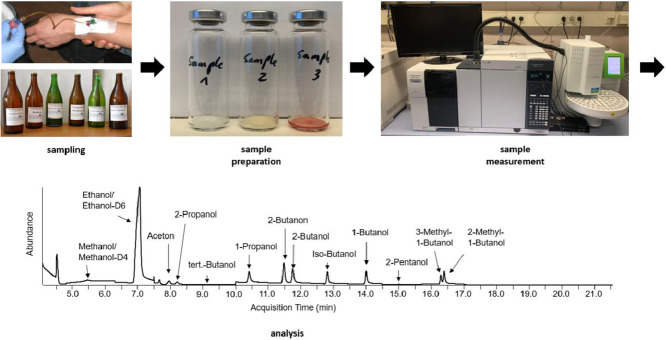

Specifications table

**Table:** 

Subject Area	Pharmacology, Toxicology and Pharmaceutical Science Pharmacology, Toxicology and Pharmaceutical Science
More specific subject area	*Forensic Toxicology*
Method name	*Analysis of ethanol and congener alcohols in serum and beverages*
Name and reference of original method	*Not applicable*
Resource availability	*Not available*

## Method details

The forensic analysis of ethanol and congener alcohols in blood is mainly performed using HS-GC-FID techniques [1]. A sensitive HS-GC-MS method was developed to detect concentrations of ethanol (g/l) beside congener alcohols, which are present in markedly lower concentrations (mg/l). By mass spectrometry, specificity was improved compared to FID-methods and interference due to matrix effects was drastically reduced. Furthermore, the two positional isomers 3-methyl-1-butanol and 2-methyl-1-butanol were adequately separated, a common problem in HS-GC-FID methods.

### Chemicals and reference substances

Water (HPLC-grade), 1-propanol, 2-propanol and 2-pentanol were purchased from VWR (Darmstadt, Germany) and was used to prepare calibration and internal standard solutions. Ethanol, ethanol-D6, methanol-D4, 2-methyl-1-butanol, and sodium sulfate (anhydrous, ≥ 90% purity) were purchased from Sigma Aldrich (St. Louis, USA), isobutanol, 2-butanone, 2-methyl- and 3-methyl-1-butanol from AppliChem (Darmstadt, Germany), methanol, 1-butanol, 2-butanol, acetone and tert.-butanol from Merck (Darmstadt, Germany). All chemicals were of analytical grade.

Serum (human/pig) was evaporated to dryness (Eppendorf® Concentrator Plus, Eppendorf, Hamburg, Germany) and redissolved with water (HPLC-grade) before analysis to guarantee congener free serum.

### Calibration standards and quality controls

A stock solution containing ethanol (100 g/l), methanol (1 g/l), 1-propanol (100 mg/l), isobutanol (2-methyl-1-propanol, 100 mg/l), 1-butanol (100 mg/l), 2-butanol (100 mg/l), 2-butanone (methyl ethyl ketone, 100 mg/l), 2-methyl-1-butanol (100 mg/l), 3-methyl-1-butanol (100 mg/l) in water was prepared and diluted for calibration standards and quality control procedures. As internal standard (ISTD), a solution containing ethanol-D6 (100 mg/l), methanol-D4 (3,2 mg/l), tert-butanol and 2-pentanol (1 mg/l, each) in water was used.

### Sample preparation

Into a 20 ml-vial containing 0.2 g sodium sulfate, 0.25 ml serum (after centrifugation of whole blood at 1900 x g for 10 min) or an alcoholic beverage sample and 25 µl ISTD solution were added. The addition of sodium sulfate increases the vapour pressure of volatile compounds present in the samples. Beverage samples were diluted with water (sample:water 1:9 and 1:99, respectively). The vial was tightly closed and vortexed. Analysis was performed in duplicate according to guidelines of GTFCh [2].

### Preparation of standards and quality controls (QC)

The final concentrations of calibration standards and low and high QC samples are presented in Table 1.

**Table 1:** Concentrations of ethanol [g/l] and congener alcohols [mg/l] in calibration (Cal) and quality control samples (QC).

Compound	Cal 1	QC 1	Cal 2	Cal 3	Cal 4	QC 2	Cal 5	Cal 6	Cal 7
Ethanol	0.10	0.25	0.50	1.0	1.5	2.0	2.5	3.0	4.0
Methanol	1.0	2.5	5.0	10	15	20	25	30	40
1-Propanol	0.10	0.25	0.50	1.0	1.5	2.0	2.5	3.0	4.0
Isobutanol	0.10	0.25	0.50	1.0	1.5	2.0	2.5	3.0	4.0
1-Butanol	0.10	0.25	0.50	1.0	1.5	2.0	2.5	3.0	4.0
2-Butanol	0.10	0.25	0.50	1.0	1.5	2.0	2.5	3.0	4.0
2-Butanone	0.10	0.25	0.50	1.0	1.5	2.0	2.5	3.0	4.0
2-Methyl-1-Butanol	0.10	0.25	0.50	1.0	1.5	2.0	2.5	3.0	4.0
3-Methyl-1-Butanol	0.10	0.25	0.50	1.0	1.5	2.0	2.5	3.0	4.0

### Analytical procedures

For the development and validation of the method, the Agilent 7890B gas chromatograph coupled to an Agilent 5977B single quadrupole mass spectrometer (Agilent Technologies, Palo Alto, USA) equipped with a Turbomatrix 110 Trap headspace sampler (Perkin Elmer, Rodgau, Germany) was used. The incubation temperature of the samples was maintained at 70 °C with an equilibration time of 20 min. The injector needle was kept at 90 °C and injection time was 0.15 min at a pressure of 135 kPa. Pressure was built-up during 0.9 min with a dwell time of 0.8 min. The temperature of the transfer line was maintained at 100 °C, that of the injector port at 130 °C in the splitless injection mode at a pressure of 95 kPa. Septum purge flow was 3 ml/min. Separation of the compounds was achieved using a VF624MS capillary column (60 m, 0.32 mm, 0.18 µm, Factor four, Agilent, California, USA). Helium was used as a carrier gas at a constant pressure of 95 kPa. The initial oven temperature of 40 °C was held for 5 min, followed by a temperature increase of 9 °C/min to a final temperature of 180 °C, which was held for 1 min. Total run time was 21.55 min for each sample. Electron impact ionisation (EI) was applied at 70 eV. Temperatures of the ion source, the quadrupole, and the interface were set at 230 °C, 150 °C and 200 °C, respectively. Chromatograms of the samples were recorded in full scan mode from 14 to 100 m/z at a solvent delay of 4.0 min, at a frequency of 7.4, and cycle time of 135 ms. For quantitation, the mass spectrometer was operated in selected ion monitoring (SIM) mode Dwell times, gain factor and m/z data are shown in Table 2, the target and qualifier ions used for quantitation in Table 3.

**Table 2:** Dwell times, gain factors and m/z of ethanol and the congener compounds.

Compound	Total Dwell time	Gain factor	Ions in group m/z
Methanol, Methanol-D4	270	1	15.1; 28.1; 29.1; 31.1; 32.1; 33.1; 34.1; 35.1; 36.1
Ethanol, Ethanol-D6	210	0.5	27.1, 28.1; 31.1; 32.1; 33.1; 41.1, 43.1; 45.1; 46.1, 49.1, 51.1
tert.-Butanol	90	1	41.1; 43.1; 55.1, 57.1; 59.1
1-Propanol	180	25	27.1; 29.1; 31.1; 42.1; 59.1; 60.1
2-Butanone, 2-Butanol	180	25	31.1; 43.1; 45.1; 57.1; 59.1; 72.1
Isobutanol	150	25	31.1; 41.1; 42.1; 43.1; 74.1
1-Butanol	150	25	31.1; 41.1; 42.1; 43.1; 56.1
2-Pentanol	90	4	45.1, 55.1; 73.1
2-/3-Methyl-1-butanol	180	25	29.1; 41.1; 42.1, 55.1; 57.1; 70.1

**Table 3:** Target and qualifier ions of ethanol and the congener compounds (Fig. 1).

Compound	Target [m/z]	Qualifier 1 [m/z]	Qualifier 2 [m/z]
Methanol-D4	35.1	33.1	
Methanol	31.0	29.0	15.0
Ethanol	46.1	45.1	31.1
Ethanol-D6	49.1	51.1	
2-Pentanol	45.1	55.1	73.1
tert.-Butanol	59.1	43.1	57.1
1-Propanol	31.1	42.1	59.1
Isobutanol	41.1	42.1	43.1
1-Butanol	56.1	41.1	43.1
2-Butanone	72.1	43.1	57.1
2-Butanol	45.1	59.1	31.1
3- Methyl-1-Butanol	42.1	55.1	70.1
2-Methyl-1-Butanol	70.1	55.1	41.1

### Quantitation parameters

Data evaluation was performed using the Agilent MassHunter Software (B.10.0). For identification, the limit of variation regarding the expected retention time compared to standard was ± 0.1 min. A quantifier/qualifier ratio within 20% of the ratio measured with calibrators were required.

## Method validation

Validation was performed according to current guidelines [3]. For statistical evaluation, Valistat 2.0 software (Arvecon GmbH, Walldorf, Germany) was used. Selectivity was assessed with serum and water samples with and without addition of internal standards (blank and zero samples). Interference by volatile substances was assessed by analysis of serum and water samples spiked with 15 volatile compounds (acetaldehyde, carbon tetrachloride, chloroform, dichloromethane, diethyl ether, 1-chlorobutane, ethyl acetate, halothane, heptane, hexane, methyl acetate, potassium cyanide, trifluoroacetic acid, toluene, xylene). Linearity was assessed by analysing seven calibration levels (each level *n* = 6) ranging from 0.1 to 4.0 g/l (ethanol), 1–40 mg/l (methanol) and 0.1–4.0 mg/l (congener alcohols) in serum and water. Sensitivity was evaluated in terms of limit of detection (LOD) and lower limit of quantification (LLOQ) using data obtained applying five or more calibrators in a low concentration range as described in the guidelines of the German Institute for Standardization [4]. For evaluation of precision and accuracy, low and high QC samples were used. QC samples were analyzed daily in duplicate during 8 days. Mean concentrations and coefficients of variation (CV) were applied for the determination of intra‐day and inter‐day precision and accuracy (bias, deviation of measured from spiked concentration). CVs and a bias below 15% were regarded as acceptable according to forensic validation guidelines [5].

## Results of method validation

**Fig. 1:**
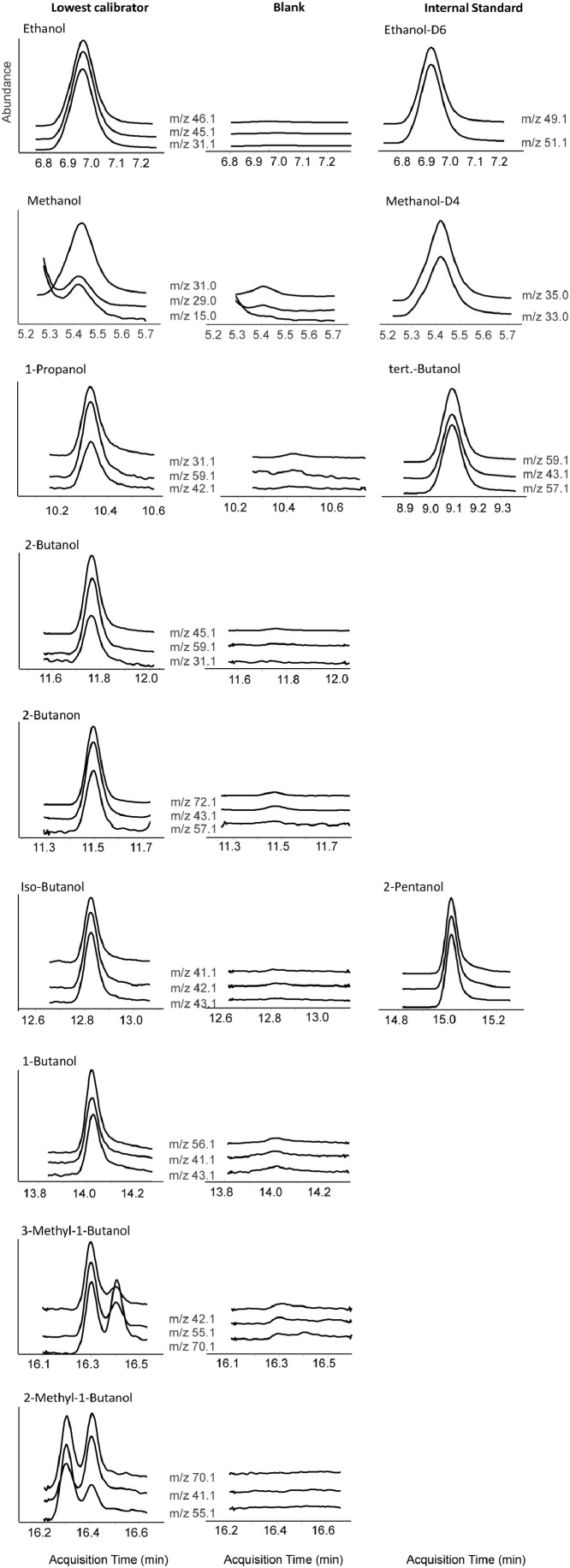
Extracted ion chromatograms of target and qualifier ions of ethanol and the congener compounds with the corresponding internal standard in the lowest calibrator (Table 1) in comparison to a blank sample (abundance in equal scale).

No interfering signals of endogenous compounds or of other volatile substances eventually present in forensic blood samples were observed (c.f. blank sample in Fig. 1). The validation data obtained were found to be within acceptable limits. LLOQs were sufficiently low and calibration curves were shown to be linear in the relevant ranges. The linear correlation for all compounds was ≥ 0.99. Intra‐day and inter‐day precision values as well as accuracies were lower than 15 % (Tables 4, 5).

**Table 4:** Validation results of ethanol and congener alcohols in serum.

Compounds	Linearity [mg/l]	LLOQ (LOD) [mg/l]	QC conc. [mg/l]	Intra-day precision [RSD%]	Inter-day precision [RSD%]	Accuracy [Bias%]
Ethanol	0.1–4.0 g/l	0.028 g/l	0.25 g/l	4.6	9.2	-1.9
		(0.0031 g/l)	2.0 g/l	3.3	6.0	-1.8
Methanol	1–40	0.23	2.5	5.5	8.9	4.8
		(0.029)	20	2.6	5.3	-2.5
1-Propanol	0.1–4.0	0.030	0.25	4.2	8.9	-5.4
		(0.0058)	2.0	4.9	6.0	-8.4
Isobutanol	0.1–4.0	0.019	0.25	5.8	6.1	-2.7
		(0.0050)	2.0	6.0	8.2	-1.6
1-Butanol	0.1–4.0	0.025	0.25	5.2	9.5	-4.1
		(0.0064)	2.0	6.2	7.6	-3.3
2-Butanol	0.1–4.0	0.026	0.25	4.3	11	-3.7
		(0.0012)	2.0	3.2	4.9	-8.8
2-Butanone	0.1–4.0	0.030	0.25	4.5	6.8	-12
		(0.0054)	2.0	3.7	4.3	-9.7
3-Methyl-1-butanol	0.1–4.0	0.020	0.25	8.3	8.8	-2.8
		(0.0078)	2.0	3.4	6.7	-3.1
2-Methyl-1-butanol	0.1–4.0	0.034	0.25	3.1	9.3	-7.9
		(0.0022)	2.0	4.1	4.9	-9.7

**Table 5:** Validation results of ethanol and congener alcohols in water.

Compounds	Linearity [mg/l]	LLOQ (LOD) [mg/l]	QC conc. [mg/l]	Intra-day precision [RSD%]	Inter-day precision [RSD%]	Accuracy [Bias %]
Ethanol [g/l]	0.1–4.0 g/l	0.028 g/l	0.25 g/l	4.5	5.8	-6.2
		(0.0034 g/l)	2.0 g/l	4.1	4.1	-4.2
Methanol	1–40	0.10	2.5	2.7	6.2	-5.7
		(0.037)	20	2.2	4.7	-5.6
1-Propanol	0.1–4.0	0.023	0.25	3.4	3.6	-9.6
		(0.0052)	2.0	2.0	6.9	-4.7
Isobutanol	0.1–4.0	0.030	0.25	6.5	7.2	-3.1
		(0.0042)	2.0	4.6	7.3	-0.04
1-Butanol	0.1–4.0	0.022	0.25	6.6	8.2	-12
		(0.0035)	2.0	6.2	8.2	-3.6
2-Butanol	0.1–4.0	0.020	0.25	2.9	4.1	-12
		(0.0045)	2.0	3.9	5.5	-9.4
2-Butanone	0.1–4.0	0.033	0.25	3.9	4.6	-15
		(0.0056)	2.0	3.5	6.0	-8.5
3-Methyl-1-butanol	0.1–4.0	0.024	0.25	5.1	6.9	-7.2
		(0.0035)	2.0	4.5	8.4	-3.9
2-Methyl-1-butanol	0.1–4.0	0.026	0.25	6.0	6.0	-14
		(0.0044)	2.0	2.5	5.0	-8.8

## Additional information

Traffic offences involving hit-and-run driving are often committed under the influence of alcohol. In these cases, the suspect sometimes claims that he/she was not under the influence of alcohol at the time of the accident, but consumed alcohol after the incident. This allegation can be proved by a congener alcohol analysis, when information on the drinking pattern (quantity and type of alcoholic beverages) is provided. This analysis was developed in the 1980s by Bonte [6]. It is based on the fact that beside ethanol, congeneric volatile compounds are present in alcoholic beverages, which are also found in blood, such as methanol, 1-propanol, iso-butanol, 1-butanol, 2-butanol and its metabolite 2-butanone, and the two positional isomers 3-methyl- and 2-methyl-1-butanol. Bonte [6] described the kinetics of the congener alcohols in blood and established formulae, which provide clues to predict concentrations in blood supernatant of congener alcohols such as in allegations made by a suspect driving under the influence of alcohol.

## Declaration of Competing Interest

The authors declare that they have no known competing financial interests or personal relationships that could have appeared to influence the work reported in this paper.
